# Metabonomic Profile of Macrosteatotic Allografts for Orthotopic Liver Transplantation in Patients With Initial Poor Function: Mechanistic Investigation and Prognostic Prediction

**DOI:** 10.3389/fcell.2020.00826

**Published:** 2020-08-28

**Authors:** Zhengtao Liu, Hai Zhu, Wenchao Wang, Jun Xu, Shuping Que, Li Zhuang, Junjie Qian, Shuai Wang, Jian Yu, Feng Zhang, Shengyong Yin, Haiyang Xie, Lin Zhou, Lei Geng, Shusen Zheng

**Affiliations:** ^1^Division of Hepatobiliary and Pancreatic Surgery, Department of Surgery, First Affiliated Hospital, School of Medicine, Zhejiang University, Hangzhou, China; ^2^NHC Key Laboratory of Combined Multi-organ Transplantation, Key Laboratory of the Diagnosis and Treatment of Organ Transplantation, CAMS, First Affiliated Hospital, School of Medicine, Zhejiang University, Hangzhou, China; ^3^Key Laboratory of Organ Transplantation, First Affiliated Hospital, School of Medicine, Zhejiang University, Hangzhou, China; ^4^DingXiang Clinic, Hangzhou, China; ^5^Shulan Hospital (Hangzhou), Hangzhou, China

**Keywords:** macrosteatosis, metabonomic, mechanism, prognosis, liver transplantation

## Abstract

**Background:**

Our previous study revealled amplified hazardous effects of macrosteatosis (MaS) on graft failure (GF) in recipients with severe liver damage in short post-operative days, with vague mechanism inside.

**Aim:**

We aimed to uncover the molecular mechanism of donor MaS on GF, and construct the predictive model to monitor post-transplant prognosis based on “omics” perspective.

**Methods:**

Ultra-performance liquid chromatography coupled to mass spectrometry metabolomic analysis was performed in allograft tissues from 82 patients with initial poor function (IPF) from multi-liver transplant (LT) centers. Pathway analysis was performed by on-line toolkit Metaboanalyst (v 3.0). Predictive model was constructed based on combinative metabonomic and clinical data extracted by stepwised cox proportional analysis.

**Results:**

Principle component analysis (PCA) analysis revealled stratification on metabolic feature in organs classified by MaS status. Differential metabolits both associated with MaS and GF were significantly enriched on pathway of glycerophospholipid metabolism (*P* < 0.05). Phosphatidylcholine (PC) and phosphatidylethanolamine (PE) involved in glycerophospholipid metabolism was significantly decreased in cases with MaS donors and GF (*P* < 0.05). Better prediction was observed on graft survival by combinative model (area under the curve = 0.91) and confirmed by internal validation.

**Conclusion:**

Metabonomic features of allografts can be clearly distinguished by MaS status in patients with IPF. Dysfunction on glycerophospholipid metabolism was culprit to link donor MaS and final GF. Decrement on PC and PE exerted the fatal effects of MaS on organ failure. Metabonomic data might help for monitoring long-term graft survival after LT.

## Introduction

Orthotopic liver transplantation (OLT) is one of the most effective therapeutic strategy for end-stage liver disease, hepatobiliary malignancy, and acute/chronic hepatic failure (Oleary et al., 2008). Donated allograft quality directly affects the patient prognosis (Feng et al., 2006; Flores and Asrani, 2017). Of which, macrosteatosis (MaS) was considered as an profound risk component for exceeded criteria donor (ECD), with positive impacts on inferior post-transplant survival and complications (Spitzer et al., 2010; Vodkin and Kuo, 2017; Moosburner et al., 2018). Followed with rising prevalence of non-alcoholic fatty liver disease (NAFLD) in donor pool, more steatotic allografts are put into clinical application to solve the organ shortage under the pressure of increasing demands for liver transplantation. Undoubtably, the influence of allograft steatosis is becoming more and more prominent for increasing proportion applied in whole patient cohort (Moosburner et al., 2018).

However, more and more concerns are raised on MaS organ for their impacts on higher comorbidities and mortality for patients after LT (Croome et al., 2019, 2020). As one of prominent sign for extended criteria donor (ECD) livers, MaS was proved to be a risk predictor on adverse prognosis including severer liver injury, increased peri-operative complications, higher post-transplant mortality and graft loss (De Graaf et al., 2012; Croome et al., 2019). In spite of controversies on acceptable safety cutoff (Liu et al., 2019), donor MaS has become the major cause for organ discard and transplant cancelation in some LT centers (Moosburner et al., 2018). Steatotic allografts deteriorate the function of transplanted liver by interaction with ischemia-reperfusion injury (Gehrau et al., 2015; Dar et al., 2019). Steatotic allografts should be transplanted under restrict control on duration of cold ischemia time (7 to 8 h) for acceptable post-transplant effects (Westerkamp et al., 2015; Wong et al., 2016).

Metabolites might be served as reliable prognostic indicator and therapeutic target for patients received LT. Baseline circulating lactate and its clearance were found to be predictor for EAD occurrence and graft survival, with better performance than conventional balance of risk (BAR) score (Golse et al., 2019; Takahashi et al., 2019).

Metabolomics data provides systematic knowledge of metabolome which might be helpful for early detection of allograft quality and prediction of prognosis after liver transplantation. And these metabolomics data can be integrated to be explained for the mechanism of inferior survival caused by clinical risk covariates, and provide potent interventions for improvement of the allograft quality (Bonneau et al., 2016; Cortes et al., 2017). Online toolkit (like MetaboAnalyst) provides concise but meaningful interpretations for the metabonomic data via pathway and enrichment analysis (Xia et al., 2015). Accordingly, prospective effects of metabolome were evaluated for prediction of early allograft dysfunction (EAD) in previous studies (Cortes et al., 2014; Xia et al., 2015). Cortes et al. emphasized the clinical value of metabonomics data on functional prediction of sub-optimal organs and donor expansion (Cortes et al., 2014). Metabolic profile was also described in grafts from donors after circulatory death (DCD) (Perera et al., 2014). However, metabolic features of MaS allografts and their connections with inferior post-transplant outcomes were still lacking.

Initial poor function (IPF), usually defined with extremely higher transminase within shorter post-operative days (PODs) (Mathe et al., 2011), plays determinative effect on post-transplant mortality and morbidity (Bolondi et al., 2016). Risk stratification was observed on post-transplant mortality and comorbidity in patients classfied by IPF occurrence (Maring et al., 1997; Hao et al., 2011). Donor MaS positively affects the IPF occurrence (Hao et al., 2011). But more importantly, MaS amplified the risk of inferior prognosis with additive on IPF (Mccormack et al., 2011). Results from our previous study found MaS allografts had worse tolerance in patients experienced IPF. Disproportionate increment on graft failure (GF) was observed by MaS allografts in patients with severe liver damage in early PODs (44% vs. 10%) (Liu et al., 2020). However, mechanism under the mortality gap is still unclear and worthy for further elucidation.

Therefore, as continuation and sublimation of previous results, we performed a multi-center study to build the predictive model for post-transplant prognosis and investigate the mechanistic link from donor MaS to GF based on combination of clinical and metabonomic indicators in patients with IPF after LT. In accordance with development of machine perfusion (MP) for organ perservation (Nasralla et al., 2018), this study provided prospective knowledge for better assessment of MaS grafts with provision of meaningful metabolites as potential targets for further improvement of allograft quality.

## Materials and Methods

### Study Flow Diagram

Procedure of study flowchart can be shown in Supplementary Figure S1. In general, allograft metabolomic, clinical and prognostic information were collected in LT cases, respectively. Donor metabolites with difference between MaS and non-MaS groups were collected (candidate metabolites A). Meanwhile, univariate survival analysis was performed for potential metabolic and clinical candidates (B and C). Then, the shared metabolites between candidate A and B were collected for further mechanistic investigation on link from allograft MaS to GF.

Meanwhile, risk model for prognostic prediction was fitted by multi-covariate analysis with inclusion of potential clinical and metabonomic factors after optimization by lasso regression. And details of procedure can be shown in Supplementary Figure S1B.

### Enrollement of Study Population

Liver transplant cases were reviewed and enrolled in the period from January 1, 2015 to March 31, 2019 from two independent transplant centers (Shulan (Hangzhou) Hospital [cohort A] and The First Affiliated Hospital of Zhejiang University [cohort B]) in accordance with uniform selection criteria as follows: (1) adults recipients (age ≥ 18 years); (2) non-living donor liver transplantation (LDLT); (3) non-multi-organ transplantation (*n* = 1); (4) occurrence of initial poor function (IPF) with definition on consecutive ALT and AST elevation within POD3 (>1500 IU/L) after liver transplantation; (5) availability of graft tissue samples kept during transplantation; 6. availability of survival status in the end of follow-up duration. Informed consents were obtained from enrolled participants. And this study was performed in accordance with the Declaration of Helsinki and approved by the ethical board of local hospital.

### Definition of Complication

Early allograft dysfunction (EAD) was diagnosed in patients with severe liver damage (ALT > 3000 IU/mL or AST > 6000 IU/mL), Jaundice (TB ≥ 10 mg/dL), and coagulation dysfunction (INR ≥ 1.6) simultaneously within POD7.

### Data Collection and Follow-Up

Clinical data related to recipients, donor, surgery and grafts was collected by experienced surgeons (ZTL and JX) respectively in local medical record system (Table 1). Graft steatosis was assessed qualitatively and quantitatively based on hematoxylin and eosin (H&E) stained sections with biopsies according to previous definition (Crowley et al., 2000). Follow-up information was collected by regular telephone call by specialized staff per month. And data on survival status, duration or death cause was provided in the end of follow-up duration.

**TABLE 1:** Summary of Clinical Information for Transplant Cases Categorized by Allograft MaS status.

Covariates	MaS grafts	Non-MaS grafts	*p*-value^a^
Number (%)	35 (42.7)	47 (57.3)	NA
**Recipient factor (R)**			
Age (R, years)	49 (34−54)	50 (43−56)	0.12
Gender (R, M,%)	30 (85.7)	39 (83.0)	0.74
BMI (R, kg/m^2^)	23.1 ± 2.7	23.8 ± 3.2	0.34
Blood Type (R)			0.98
A-type n (%)	15 (42.9)	18 (38.3)	
B-type n (%)	4 (11.4)	6 (12.8)	
O-type n (%)	14 (40)	20 (42.6)	
AB-type n (%)	2 (5.7)	3 (6.4)	
Diabetes (R, N,%)	3 (8.6)	7 (14.9)	0.39
Pre-operative AFP (R, ng/ml)	30.4 (4.9−551.4)	16.1 (5.6−139.0)	0.44
HBV infectors (R, N,%)	24 (68.6)	39 (83.0)	0.13
MELD score (R)	33 (28−40)^∗^	33 (26−40)	0.70
Child–pugh score (R)	10 (9−11)	11 (10−12)	0.11
**Donor factor (D)**			
Age (D, years)	45 (31−51)	44 (36−53)	0.80
Gender (D, M,%)	29 (82.9)	40 (85.1)	0.78
BMI (D, kg/m^2^)	23.8 ± 2.8	22.9 ± 2.5	0.13
Blood type (D)			0.60
A-type n (%)	13 (37.1)	14 (29.8)	
B-type n (%)	5 (14.3)	7 (14.9)	
O-type n (%)	13 (37.1)	19 (40.4)	
AB-type n (%)	4 (11.4)	7 (14.9)	
HBV infectors (D, N,%)	6 (17.1)	5 (10.6)	0.39
HCV infectors (D, N,%)	6 (17.1)	0 (0)	NA
**Pre-donation blood test (D)**			
D-Potassium (mmol/L)	3.7 (3.4−4.1)	4 (3.7−4.6)	0.02
D-Sodium (mmol/L)	145.9 (139.0−152.0)	145.8 (138.1−153.1)	0.79
D-ALT (U/L)	44.0 (25.0−74.0)	39.4 (25−62)	0.55
D-TB (μmol/L)	14.8 (10.4−21.4)	19.3 (11−27)	0.15
D-CR (μmol/L)	87.0 (55.0−160.0)	86.3 (61.0−151.6)	0.82
D-BUN (mmol/L)	7.6 (5.5−10.9)	8.6 (5.0−11.6)	0.57
Donation type (DBD/DCD/DBCD)			0.25
DBD (N, %)	12 (34.2)	10 (21.3)	
DCD (N, %)	16 (45.7)	30 (63.8)	
DBCD (N, %)	7 (20.0)	7 (14.9)	
Cause of Death (TBI/Stroke/Others)	17/16/2	23/20/3	0.98
ECMO use	0	0	NA
**Graft factor (G)**			
Steatosis Severity (%)	15 (5−25)	10 (5−18.8)	<0.01
CIT (min)	646 (542−744)	652 (567−743)	0.68
WIT (min)	5 (0−10)^∗^	9 (5−12)	0.03
**Surgery (S)**			
Indication for LT			0.65
Liver Cirrhosis n (%)	13 (37.1)	22 (46.8)	
Liver Failure n (%)	10 (28.6)	7 (14.9)	
PBC/PSC n (%)	2 (5.7)	2 (4.3)	
Liver Cancer n (%)	17 (48.6)	19 (40.4)	
Others n (%)	1 (2.9)	2 (4.3)	
Post-LT Peak TB level (mg/dL)	205.9 (106−386)	225 (138−387)	0.58
Post-LT Peak ALT Level (IU/L)	2626 (2027−3694)	2401 (1972−3075)	0.35
Post-LT Peak AST level (IU/L)	6576 (4673−13638)	6049 (3665−8745)	0.27
EAD occurrence n (%)	22 (62.9)	30 (63.8)	0.93
PNF occurrence n (%)	4 (11.4)	6 (12.8)	0.86
Blood Transfusion during LT	745 (630−1220)	775 (510−1020)	0.65
pRBC (U)	4.5 (2.0−8.0)	5.0 (2.0−9.0)	0.71
FFP (ml)	3000 (0−3500)	3000 (1500−4000)	0.48
PCC (U)	2000 (0−3000)	1750 (75−3000)	0.19
FIB (g)	5 (0−7.5)	5 (2−10)	0.44
ALB (g)	115 (30−150)	125 (75−150)	0.50
Blood Loss (ml)	1500 (1000−2500)	1200 (800−2000)	0.12
Surgical Duration (mins)	310 (275−375)	302.4 (260−339)	0.40
ICU stay (days)	12.8 (7.6−17)	13 (7.6−18)	0.97
Length of post−LT hospitalization (d)	29 (19−39)	26 (12−37)	0.72
**Year of LT**			0.07
2015−2016 (n,%)	7 (20)	20 (42.6)	
2017−2019 (n,%)	28 (80)	27 (57.4)	
Time from LT to the end of follow-up survey (days)	616 (510−885)	894 (624−1670)	0.02

*Represented significant difference across different groups; a represented. ^a^comparison was performed by one-way ANOVA for quantitative data in symmetrical distribution; by Mann−Whitney U-test for quantitative data in asymmetrical distribution; by Chi-square test for count data. D, donor; DBCD, donation after brain and cardiac death; DBD, donation after brain death; DCD, donation after cardiac death; EAD, early allograft dysfunction; ECMO, extracorporeal membrane oxygenation; FFP, fresh frozen plasma; FIB, fibrinogen; G, graft; HBV, hepatitis B virus; HCV, hepatitis C virus; ICU, intensive care unit; LT, liver transplantation; M, male; MaS, macrosteatosis; MELD, model for end-stage liver disease; PBC, primary biliary cholangitis; PCC, prothrombin complex concentrate; PNF, primary non-function; PSC, primary sclerosing cholangitis; R, recipient; RBC, red blood cell; TB, total bilirubin; TBI, traumatic brain injuries.

### Sample Collection and Preparation

Graft tissues were routinely collected from grafts for transplantation after their reperfusion in perfusates. Samples were flash frozen in liquid nitrogen once seperated from allografts and kepted rountinely in ultra-low temperature freezer (−80°C) in biobank of NHC Key Laboratory of Combined Multi-organ Transplantation for long-term storage. Samples were accurately weighted and extracted in solvent ethanol/water mixture with internal reference for further metabolomic analysis. And details of the treatment can be shown in Supplementary Material.

### Ultra-Performance Liquid Chromatography Coupled to Mass Spectrometry (LC-MS) Metabolomics

Profile of metabolites were tested by Dionex Ultimate 3000 RS UHPLC system (Thermo Fisher Scientific) with heated electrospray ionization in positive and negative modules. Potential metabolites was obtained and identified by progenesis QI software (Waters Corporation), based on Human Metabolome Database (HMDB^1^). QC samples were injected every 10 samples for accessible repeatability. Details of the parameters in sample detection and data process can be referred to Supplementary Material.

### Network and Pathway Analysis

Based on SIMCA-P platform (Wu et al., 2010) (version 14.1, Umetrics, Sweden), principle component analysis (PCA) and orthogonal partial least-squares-discriminant analysis (OPLS-DA) were carried out to present metabolic alterations across MaS and non-MaS groups. Variable importance in the projection (VIP) value was calculated for each covariate, and VIP > 1 was indicative of relevance with group discrimination. Enrichment and pathway analysis on metabolomic data were conducted based on Kyoto Encyclopedia of Genes and Genomes (KEGG) database were performed and visualized by MetaboAnalyst software (version 4.0^2^) for deeper knowledge of biological connection across potential metabolites (Xia and Wishart, 2016).

### Statistic Analysis

Normally distributed data was described as mean ± standard deviation (SD) and compared by one-way ANOVA. Abnormally distributed data was presented as median (inter-quartile range, IQR) and compared by Mann-Whitney U test. Categorical data was presented as number (percentage) and compared by chi-square test.

For survival analysis, cox proportional-hazards regression model was used for selection of prognostic factors. Lasso regression was used to select the optimal prognostic covariates by reduction of the high dimensional data (Friedman et al., 2010). Potential covariates for predicative model was filtered using multivariable cox regression model adjusted by optimized factors. Correlations across significant indicators were evaluated by spearman heatmap.

For single predictor, the Kaplan-Meier curves were plotted to show its dichotomous effect on overall survival; and two-stage random effect model was used for evaluation on its dose-response association with prognosis (Orsini et al., 2006).

Predictive nomogram was plotted based on covariates from multivariable cox regression analysis. C-statistic was used to quantitatively evaluate the discriminative performance of nomogram (Pencina and Dagostino, 2004). And calibration curves were plotted to reflect the agreement between actual outcomes and predicted probabilities (Kramer and Zimmerman, 2007). Receiver operating characteristic (ROC) curve analysis was conducted to evaluate the predictive performance of clinical, metabonomic and combinative clusters on GF. Diagnostic performances of these clusters were assessed using the area under the receiver operating curve (AUROC) and discriminated by z statistic analysis. Time-dependent AUROC was also plotted to evaluate the performance of these clusters in prediction of GF in different period (Hung and Chiang, 2009).

Statistic analysis was performed via R (v.3.5.1), stata (v.14.0), SPSS (v.26.0), medcalc (v.19.0.7), respectively. Details of software and algorithm were shown in Supplementary Table S1. Two-sided *P*-value < 0.05 was considered as statistical significance.

## Results

### Clinical Characteristics of Enrolled Patients

Patients were included with severe post-transplant liver damage (ALT ≥ 2000 IU/L), based on cohorts of 975 LT cases. In contrast to the stable tendency on utilization MiS liver, donation of MaS allografts was significantly increased from 15% in 2015 to more than one quarter in 2019 (*P* < 0.05, Supplementary Figure S2). Selection procedure can be shown in Supplementary Figure S2. Eighty-two patients with post-transplant IPF were enrolled into final analysis. As shown in Table 1, the MaS prevalence was about 42.6% in grafts received metabonomic analysis. Insignificant difference was observed in patients categorized by allograft steatosis in most dimensions (*P* > 0.05). Positive anti-HCV was only observed in MaS but not in non-MaS grafts. WIT was shorter in patients using MaS grafts for LT (*P* < 0.05). Intriguingly, the follow-up duration was nearly a third shorter in donor MaS group (616 vs. 786 days, *P* < 0.05), verifying the increasing trend on utilization of MaS allografts in the whole cohort. Insignificant difference was observed distribution of patients’ status (age, gender, blood type, etc.) and disease severity (Child-Pugh/MELD score) in groups categorized by medical centers (*P* > 0.05, Supplementary Table S2).

### Predictive Clinical Factors on Graft Failure

**FIGURE 1:**
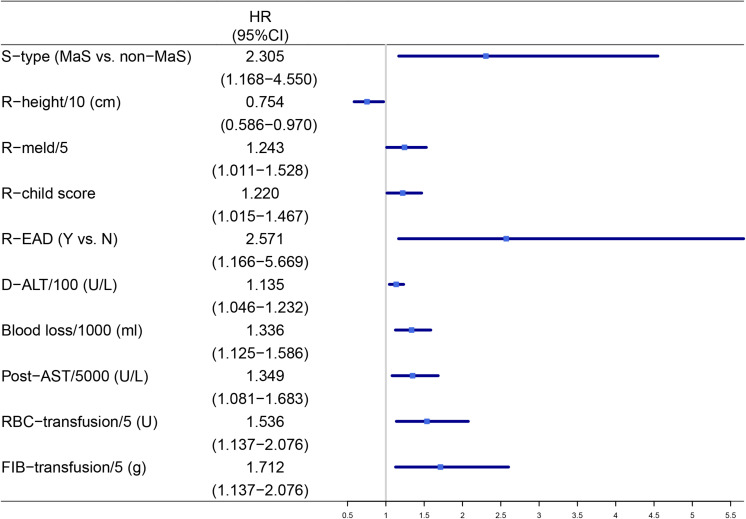
Factors significantly associated with post-transplant graft failures after multi-covariate analysis. AST, aspart aminotransferase; D, donor; EAD, early allograft dysfunction; FIB, fibrinogen; HR, hazard ratio; MaS, macrosteatosis; R, recipient; RBC, red blood cell; S, steatosis.

Clinical factors on recipient (pre-operative child-pugh/MELD score, height, postoperative AST level), donor (pre-operative ALT), graft (macrosteatosis) and surgical (blood loss/transfusion) aspects commonly affected the graft survival in multi-covariate cox proportional hazard model (Figure 1). Noteworthy, significantly higher risk of GF was observed in patients with EAD occurrence and MaS graft utilization (HR = 2.57/2.30, *P* < 0.05).

### Metabonomic Profiles of Donor Livers

Raw data was adjusted by QC samples according to predefined criteria (Sangster et al., 2006). A total of 3444 metabolites were detected per sample after data pretreatment by Progenesis QI (v2.3). Finally, 2155 features with identification in Human Metabolome Database^3^ were enrolled for further analysis.

### Multivariate Analysis (MVA) on Donor Livers

**FIGURE 2:**
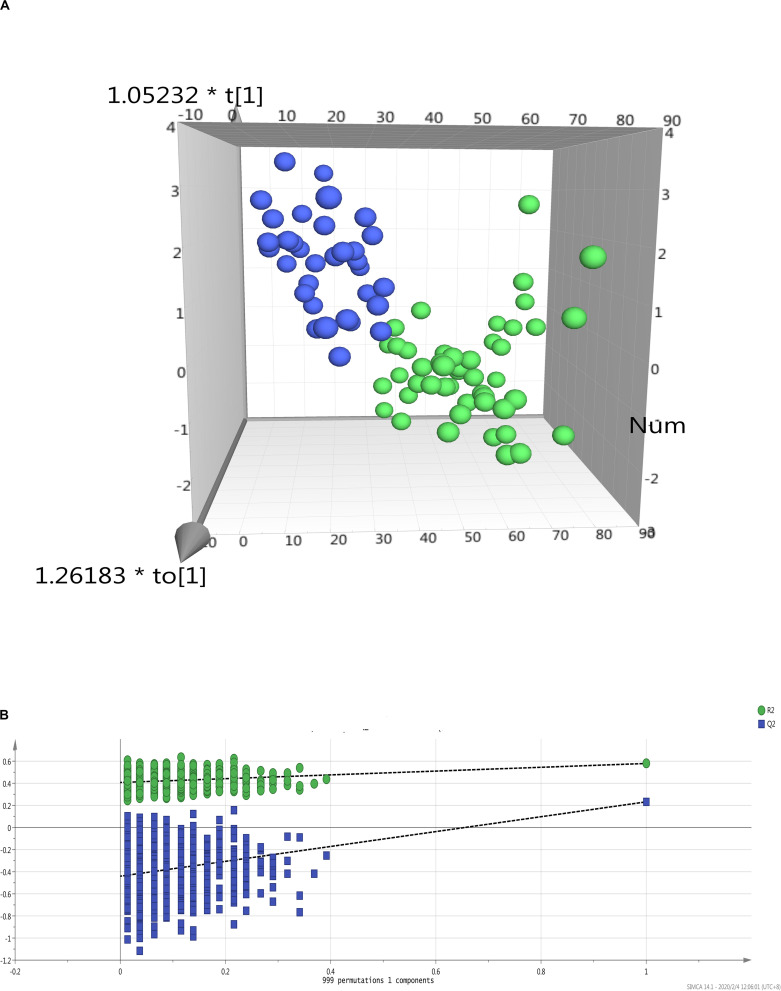
Multivariate data analysis on metabolic profiles of donor livers by MaS status. **(A)** PCA analysis revealled clear separation on patients received MaS (blue dots) and non-MaS (green dots) allografts by OPLS-DA model; **(B)** Validation of OPLS-DA model by class permutation analysis for panel **(A)**. MaS, macrosteatosis, OPLS-DA, orthogonal projection to latent structures discriminant analysis; PCA, principal component analysis.

Multivariate analysis in OPLS-DA model revealed clear separation on metabonomic features between MaS and non-MaS grafts (Q^2^ = 0.58, R^2^ = 0.52, Figure 2A). Further validation model by permutation test also showed the specificity and reliability of the patient classification (R^2^ = 0.41, Q^2^ = −0.441, Figure 2B).

### Network Analysis on Potential Metabolites Associated With Donor MaS and Graft Failure

**FIGURE 3:**
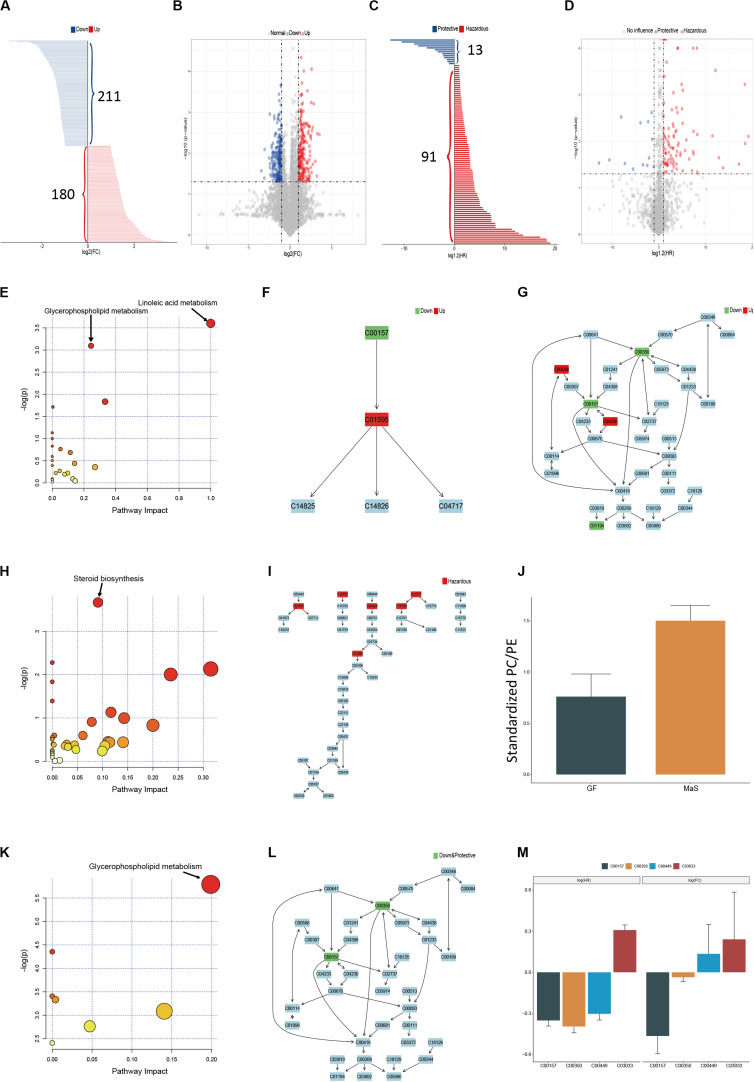
Pathway enrichment based on metabolites associated withdonor MaS, graft failure and their intersection. **(A)** Bar chart discriminating the components with significant increments (red bar, *n* = 180) or decrement (blue bar, *n* = 211) in MaS grafts; **(B)** Volcano plot on visualization of both FC and significance for each metabolites compared between MaS and non-MaS grafts, red dots represented significantly higher metabolits (FC > 2, *P* < 0.05) in MaS grafts, blue dots represented significantly lower metabolits (FC < 0.5, P < 0.05) in MaS grafts; **(C)** Bar chart discriminating the components with significant hazardous (blue bar, *n* = 13) or protective (red bar, *n* = 91) effects on graft failure; **(D)** Volcano plot on visualization of both HR and significance for each metabolites compared between organs with and without graft failure, blue dots represented metabolites with protective effects on graft failure (HR < 0.5, *P* < 0.05), red dots represented metabolites with harzardous effects on graft failure; **(E)** Results for pathway enrichment based on metabolits with difference between MaS and non-MaS grafts, pathway on linoleic acid and glycerophospholipid metabolism were significantly associated with donor MaS (*P* < 0.05); **(F)** Details of linoleic acid metabolism pathway and related metabolites involved in donor MaS; **(G)** Details of glycerophospholipid metabolism pathway and related metabolites involved in donor MaS; **(H)** Results for pathway enrichment based on differential metabolites in univariate cox proportional analysis on graft failure, pathway on steroid biosynthesis was significantly associated with graft failure; **(I)** Details of steroid biosynthesis and related metabolites involved in graft failure; **(J)** Standardized PC/PE ratios in subgroup patients received MaS grafts or cases with graft failure occurrence; **(K)** Results for pathway enrichment based on metabolites intersective between E and H, pathway on glycerophospholipid metabolism was significantly associated with MaS related graft failure; **(L)** Details of glycerophospholipid metabolism pathway and related metabolites involved in MaS-related graft failure; **(M)** Metabolites both involved in MaS and graft failure. Green box presented down-regulation in MaS grafts and/or protective effect on graft failure; Red box represented up-regulation in MaS and/or hazardous effects on graft failure. FC, fold change; HR, hazard ratio; MaS, macrosteatosis; PC, phosphatidylcholine; PE, phosphatidylethanolamine.

Significant variation was observed across MaS and non-MaS allografts in 389 metabolites by univariate ANOVA analysis (higher in 211, but lower in 180 features for MaS grafts, Figure 3). Further functional pathway analysis revealled that the differentiated metabolites caused by MaS were mainly involved in participation of linoleic acid and glycerophospholipid metabolism (*P* < 0.05, Figure 3 and Supplementary Table S3). Compounds involved in candidate pathways for MaS allografts were reviewed in Table 2. Most of potential features can be categorized into glycerophospholipids class. Linoleic acid level was significantly higher, but phosphatidylcholine and phosphatidylethanolamine levels were decreased in MaS donors (*P* < 0.05).

**TABLE 2:** Summary of potential metabolites in candidate pathways responsible for donor MaS and graft failure.

MaS vs. Non-MaS	Structure	ID			Category			Statistics			Biological Involvement	
					
Metabolites	Formula	KEGG	HMDB	LIPID MAPS	Super Class	Main Class	Sub Class	FC/HR (95%CI)	*P*-value	Trend-a	Pathway	Function
PC(20:5/16:0)	C44H7 8NO8P	C00157	HMDB00 08495	LMGP010 11932	Lipids and lipid-like molecules	Glycero phospho lipids	Glycero phosphocholines	0.45	0.041	down	Linoleic acid/Glycero phospholipid metabolism	Known as phosphatidylcholine, consists of one chain of eicosapentaenoic acid at the C-1 position and one chain of palmitic acid at the C-2 position, involved in metabolism and signaling.
Linoleic acid	C18H3 2O2	C01595	HMDB00 00673	LMFA010 30120	Lipids and lipid-like molecules	Fatty Acyls	Lineolic acids and derivatives	2.57	0.004	up	Linoleic acid metabolism	Known as an essential fatty acid in human nutrition because it cannot be synthesized by humans. Used in the biosynthesis of prostaglandins and cell membranes. Associated with isovaleric acidemia, which is an inborn error of metabolism.
PE(20:4/22:6)	C47H74 NO8P	C00350	HMDB00 09408	LMGP020 10961	Lipids and lipid-like molecules	Glycero phospholipids	Glycerophospho ethanolamines	0.31	0.042	down	Glycero phospholipid metabolism	Also named as phosphatidylethanolamine
PE(20:5/18:2)	C43H72 NO8P	C00350	HMDB00 09456	LMGP020 10974	Lipids and lipid-like molecules	Glycero phospholipids	Glycerophospho ethanolamines	0.29	0.036	down	Glycero phospholipid metabolism	Also named as delta7- Avenasterol, as intermediate in the biosynthesis of steroids
LysoPC(20:3)	C28H5 2NO7P	C04230	HMDB00 10393	LMGP01 050139	Lipids and lipid-like molecules	Glycero phospholipids	Glycero phosphocholines	2.00	0.010	up	Glycero phospholipid metabolism	Known as glycerophosphocholines in which the glycerol is esterified with a fatty acid at O-1 position, and linked at position 3 to a phosphocholine.
LysoPC(20:4)	C28H5 0NO7P	C04230	HMDB0 010395		Lipids and lipid-like molecules	Glycero phospholipids	Glyceropho sphocholines	2.21	0.008	up	Glycero phospholipid metabolism	Known as lysophospholipids which has a role in lipid signaling by acting on lysophospholipid receptors.
LysoPC(22:4)	C30H5 4NO7P	C04230	HMDB00 10401		Lipids and lipid-like molecules	Glycero phospholipids	Glyceropho sphocholines	2.12	0.021	up	Glycero phospholipid metabolism	Known as lysophospholipids which has a role in lipid signaling by acting on lysophospholipid receptors.
LysoPC(22:5)	C30H5 2NO7P	C04230	HMDB00 10403	LMGP010 50143	Lipids and lipid-like molecules	Glycero phospholipids	Glycero phosphocholines	2.03	0.028	up	Glycero phospholipid metabolism	Known as lysophospholipids which has a role in lipid signaling by acting on lysophospholipid receptors.
Phosphocholine	C5H14 NO4P	C00588	HMDB0 001565		Organic nitrogen compounds	Organonitrogen compounds	Quaternary ammonium salts	2.71	0.012	up	Glyceropho spholipid metabolism	Known as choline phosphate, participates in a number of enzymatic reactions, can be converted into choline through its interaction with the enzyme phosphoethanolamine/phosphocholine phosphatase.
1-Phosphatidyl-D-myo-inositol	C11H1 9O13P	C01194	HMDB00 06953	LMGP06 010000	Lipids and lipid-like molecules	Glyceropho spholipids	Glyceropho sphoinositols	0.40	0.046	down	Glyceropho spholipid metabolism	Unclear
**Graft Survival**												
Calcidiol	C27H 44O2	C01561	HMDB0 003550		Lipids and lipid-like molecules	Steroids and steroid derivatives	Vitamin D and derivatives	1.67 (1.21- 2.32)	0.002	Hazardous	Steroid biosynthesis	Major circulating metabolite of vitamin D3, produced in liver and the best indicator of the body’s vitamin D stores. Effective in treatment of rickets and osteomalacia.
Delta7-Avenasterol	C29H 48O	C15782	HMDB00 06851	LMST01 040154	Lipids and lipid-like molecules	Steroids and steroid derivatives	Stigmastanes and derivatives	1.31 (1.03-1.68)	0.030	Hazardous	Steroid biosynthesis	Known as delta7-Avenasterol as intermediate in biosynthesis of steroids, converted from 24-Ethylidenelophenol, then converted to 5-dehydroavenasterol in synthesis of Stigmasterol.
Presqualene diphosphate	C30H52 O7P2	C03428	HMDB00 01278	LMPR0106 010003	Lipids and lipid-like molecules	Prenol lipids	Triterpenoids	1.27 (1.08-1.64)	0.045	Hazardous	Steroid biosynthesis	Known as presqualene diphosphate as an intermediate in the biosynthesis of terpenoid. Substrate for farnesyl-diphosphate farnesyltransferase.
Episterol	C28H46O	C15777	HMDB00 06847	LMST01 030115	Lipids and lipid-like molecules	Steroids and steroid derivatives	Ergostane steroids	1.08 (1.02-1.18)	0.044	Hazardous	Steroid biosynthesis	Involved in the biosynthesis of steroids. Converted from 24-Methylenelophenol to 5-Dehydroepisterol by lathosterol oxidase
5-Dehydroepisterol	C28H44O	C15780	HMDB00 06848	LMST01 030135	Lipids and lipid-like molecules	Steroids and steroid derivatives	Ergostane steroids	1.59 (1.05-2.66)	0.041	Hazardous	Steroid biosynthesis	As an intermediate in the biosynthesis of steroids, converted from Episterol, then converted to 24-Methylenecholesterol.
4,4-Dimethylcholesta-8,14,24-trienol	C29H46O	C11455	HMDB0 001023		Lipids and lipid-like molecules	Steroids and steroid derivatives	Cholestane steroids	1.98 (1.07-3.69)	0.031	Hazardous	Steroid biosynthesis	Involved in the biosynthesis of steroids and involved in the conversion from lanosterol to zymosterol.

Graft survival was significantly affected by 104 metabolic features using univariate cox analysis (91 hazardous and 13 protective metabolites, Figure 3). Enrichment of candidate metabolites indicated the significance of steroid biosynthesis pathway on post-transplant prognosis (*P* < 0.05, Figure 3 and Supplementary Table S4). Most involved features can be categorized into steroids class and exerted hazardous effects on post-transplant prognosis (Figure 3 and Table 2).

After classification by KEGG IDs, the compounds including phosphatidylcholine (C00157), phosphatidylethanolamine (C00350), saccharopine (C00449) and glucuronide (C03033) were overlapped metabolominc clusters with both association on post-transplant prognosis and donor MaS (Figure 3). C03033 increased both risk on MaS occurrence and GF, while the C00157 and C00350 exerted protective effects on above two events (Figure 3). Network analysis revealled the overlapped metabolites were enriched significantly on pathway of glycerophospholipid metabolism (*P* < 0.01, Figure 3 and Supplementary Table S5).

### Selection of Candidates for Prognostic Analysis

Positive clinical and metabonomic variables in prior univariate analysis were put into LASSO regression model for dimensional-reduction of the dataset. 32 factors with inclusion of 23 metabolomic and 9 clinical features were screen out for further analysis. Finally, 15 factors including 10 metabonomic and 5 clinical features were selected with most represensitivity for further predictive model for post-transplant prognosis.

### Potential Model With Cobination of Clinical and Metabonomic Signatures on Prognostic Prediction

**FIGURE 4:**
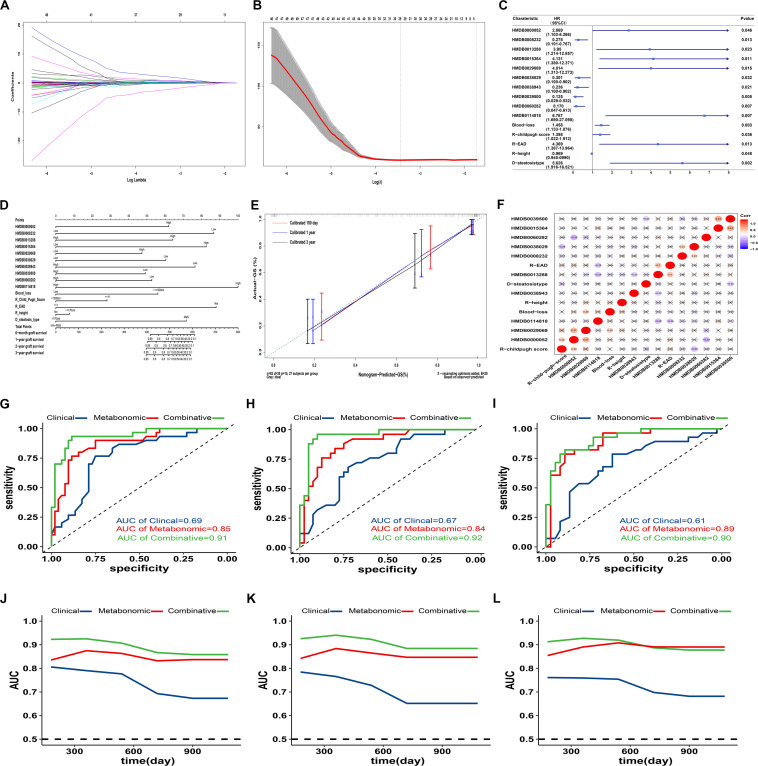
Predictive effects of clinical-metabonomic model on post-transplant prognosis. **(A)**. Lasso coefficient profiles of selected factors in univariate analysis; **(B)** Optimal parameter selection in LASSO model by cross-validation via minimum criteria. Partial likelihood deviance curve was plotted versus log(λ). Dotted vertical lines were drawn at the center of optimal values using the minimum criteria within one SE of the minimum criteria. **(C)** Forest plot of potential candidates with construction for predictive model on GF occurrence by cox proportional analysis; **(D)** Nomogram for GF prediction based on candidate clinical and metabonomic factors; **(E)** Calibration curves for association between predicted and actual GF in different time points. **(F)** Heatmap with pairwise correlation analysis across potential clinical and metabonomic covariates; **(E)** Performance of different models (clinical, metabonomic and combinative) on GF prediction in all LT cases; **(F)** Performance of different models (clinical, metabonomic and combinative) on GF prediction in LT cases from cohort A; **(G)** Performance of different models (clinical, metabonomic and combinative) on GF prediction in LT cases from cohort B; **(H)** Time-dependent AUROC values for different models on GF prediction in all LT cases; **(I)** Time-dependent AUROC values for different models on GF prediction in LT cases from cohort A; **(J)** Time-dependent AUROC values for different models on GF prediction in LT cases from cohort B; **(K)**. GF, graft failure; LASSO, least absolute shrinkage and selection operator; LT, liver transplantation; SE, standard error.

Fifteen factors with statistic significance in multi-covariate Cox regression were enrolled for construction of clinical-metabonomic predictive model for post-transplant prognosis (Figure 4). Prominently higher risk of GF was observed in patients with EAD occurrence or utilization of MaS donors (HR = 4.37/5.62, respectively). The panorama of enrolled susceptive metabolites was summarized in Table 3. Most of these metabolites can be clustered into to lipid and organic acid categories, respectively. Based on clinical-metabonomic model, the C00157 compound [PC(18:4/16:0)] exerted protective effect, while the dexamethasone (HMDB0015364) as extraneous glucocorticoid played hazardous role on inferior prognosis after LT (HR = 0.28 and 4.13, respectively).

**TABLE 3:** Summary of candidate meatbolites for predictive model on post-transplant prognosis.

	Structure	Identification			Category			Biological Involvement
Metabolites	Formula	KEGG	HMDB	LIPID MAPS	Super class	Main class	Sub class	Function
(E)-Avenanthramide D	C16H13NO4		HMDB0038943		Phenylpropanoids and polyketides	Cinnamic acids and derivatives	Hydroxycinnamic acids and derivatives	Belongs to the avenanthramides. Detected outside of the human body, in, cereals and cereal products and oats, which make (e)-avenanthramide D as potential biomarker for the consumption of these foods.
3′-UMP	C9H13N2O9P	C01368	HMDB0060282		Nucleosides, nucleotides, and analogs	Ribonucleoside 3′-phosphates	Unclassified	Also known as uridine 3′-phosphoric acid or 3′-uridylic acid, belongs to the ribonucleoside 3′-phosphates. Uridine 3′-monophosphate exists in all living organisms, ranging from bacteria to humans.
Argininosuccinic acid	C10H18N4O6	C03406	HMDB0000052		Organic acids and derivatives	Carboxylic acids and derivatives	Amino acids, peptides, and analogs	Known as a basic amino acid. Cells synthesize it from citrulline, aspartic acid and use it as a precursor for arginine in the urea cycle or Citrulline-NO cycle. As a precursor to fumarate in the citric acid cycle via argininosuccinate lyase.
Dexamethasone	C22H29FO5	C15643	HMDB0015364		Lipids and lipid-like molecules	Steroids and steroid derivatives	Hydroxysteroids	Only found in individuals have used or taken this drug. It is anti-inflammatory 9-fluoro-glucocorticoid as a glucocorticoid agonist, used for its antiinflammatory or immunosuppressive properties. Also able to penetrate the CNS, used to manage cerebral edema. Complex between Dexamethasone and cytoplasmic glucocorticoid receptors binds to DNA elements results in a modification of transcription and protein synthesis in order to achieve inhibition of leukocyte infiltration at the site of inflammation, interference in the function of mediators of inflammatory response, suppression of humoral immune responses, and reduction in edema or scar tissue. The anti-inflammatory actions of dexamethasone are thought to involve phospholipase A2 inhibitory proteins, lipocortins, which control the biosynthesis of potent mediators of inflammation such as prostaglandins and leukotrienes.
Eriojaposide B	C25H40O11		HMDB0038029		Lipids and lipid-like molecules	Fatty Acyls	Fatty acyl glycosides	Belongs to the class of organic compounds, known as fatty acyl glycosides of mono- and disaccharides.
N-Malonyltryptophan	C14H14N2O5		HMDB0 039500		Organic acids and derivatives	Carboxylic acids and derivatives	Amino acids, peptides, and analogs	Belongs to the class of organic compounds known as n-acyl-alpha amino acids. N-acyl-alpha amino acids are compounds containing an alpha amino acid which bears an acyl group at its terminal nitrogen atom. Detected outside of the human body in foods like tomato, herbs and spices, opium poppies pulses, which make it as potential biomarker for the consumption of these substance.
Non-anoylcarnitine	C16H31NO4		HMDB00 13288	LMFA07 070082	Lipids and lipid-like molecules	Fatty Acyls	Fatty esters	Classified as a member of the acyl carnitines, practically insoluble in water and weak acidic. Considered as a fatty ester lipid molecule, which can be found in blood and urine. Primarily located in the extracellular space and near the membrane.
PA(15:0/18:4)	C36H63O8P		HMDB01 14818	LMGP10 010146	Lipids and lipid-like molecules	Glyceropho spholipids	Glycerophosphates	As glycerophospholipid in which a phosphate moiety occupies a glycerol substitution site. PA(15:0/18:4(6Z,9Z,12Z,15Z)) consists of one chain of pentadecanoic acid at the C-1 position and one chain of stearidonic acid at the C-2 position. Phosphatidic acids are quite rare but are extremely important as intermediates in the biosynthesis of triacylglycerols and phospholipids.
PC(18:4/16:0)	C42H76NO8P	C00157	HMDB00 08232	LMGP01 011706	Lipids and lipid-like molecules	Glyceropho spholipids	Glyceropho sphocholines	Known as glycerophospholipid in which a phosphorylcholine moiety occupies a glycerol substitution site. Consists of one chain of stearidonic acid at the C-1 position and one chain of palmitic acid at the C-2 position. Ubiquitous in nature as key components of the lipid bilayer of cells, also being involved in metabolism and signaling. Stearidonic acid moiety is derived from seed oils, while the palmitic acid moiety is derived from fish oils, milk fats, vegetable oils and animal fats.
Threoninyl-Proline	C9H16N2O4		HMDB0 029069		Organic acids and derivatives	Carboxylic acids and derivatives	Amino acids, peptides, and analogs	Known as dipeptide composed of threonine and proline as incomplete breakdown product of protein digestion or protein catabolism. Dipeptides are known to have physiological or cell-signaling effects although most are simply short-lived intermediates on the way to specific amino acid degradation pathways following further proteolysis.

**FIGURE 5:**
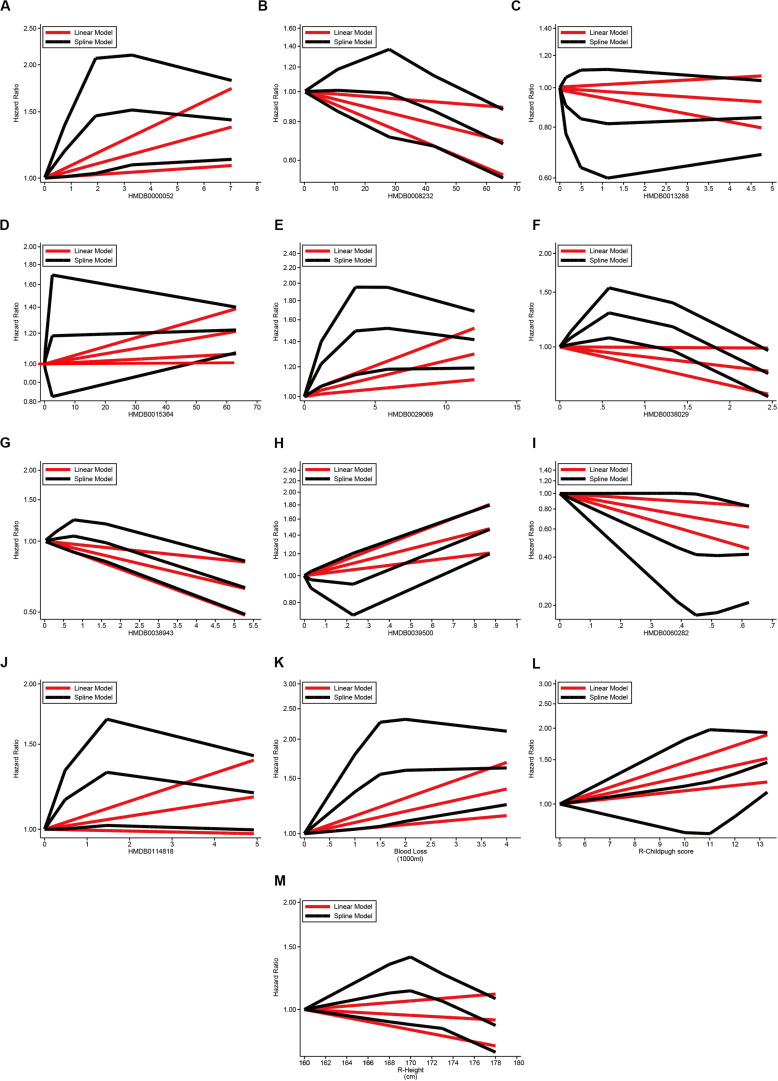
Dose-response effects of continuous covariates on graft failure via GLS and RCS models. **(A)** Dose-response effects of argininosuccinic acid (HMDB0000052) on GF; **(B)** Dose-response effect of PC(18:4/16:0) (HMDB0008232) on GF; **(C)** Dose-response effect of non-anoylcarnitine (HMDB0013288) on GF; **(D)** Dose-response effect of dexamethasone (HMDB0015364) on GF; **(E)** Dose-response effect of threoninyl-proline (HMDB0029069) on GF; **(F)** Dose-response effect of eriojaposide B (HMDB0038029) on GF; **(G)** Dose-response effect of (E)-Avenanthramide D (HMDB0038943) on GF; **(H)** Dose-response effect of N-Malonyltryptophan (HMDB0039500) on GF; **(I)** Dose-response effect of 3′-UMP (HMDB0060282) on GF; **(J)** Dose-response effect of PA(15:0/18:4) (HMDB0114818) on GF; **(K)** Dose-response effect of blood loss (per 1000 ml) on GF; **(L)** Dose-response effect of pre-transplant child-pugh score of recipients on GF; **(M)** Dose-response effect of recipient height (cm) on GF; Linearity on effects of covariates on post-transplant GF was estimated via GLS and RCS models, respectively. GF, graft failure; GLS, generalized least squares; LT, liver transplantation; RCS, restricted cubic splines.

Further dose-response analysis on each potential factors revealled that the risk trend of GF was observed consistently in linear trend in 10 clinical-metabonomic factors (P for non-linearity > 0.05, Figure 5 and Supplementary Table S6). Hazardous effects of threoninyl-proline and PA(15:0/18:4) might stay on plateau after their arrival on risk peak for GF. Compared to grafts in lowest quintile, the HR of Eriojaposide B (HMDB0038029) rose to 1.29 in Q3, but descend to 0.82 in highest quintile. As external substance, dexamethasone and N-Malonyltryptophan can’t be detected in 50% and 37% of allografts, but the GF risk was increased rapidly once tested in remaining organs.

### Nomogram for Prediction of Post-transplant Graft Failure

Fifteen factors (10 metabonomic and 5 clinical) significantly associated with GF in cox-regression model were integrated into predictive nomogram for post-transplant graft survival in different time periods (Figure 4). The concordance index for the nomogram was 0.85 (95%CI: 0.79−0.91). Calibration plot showed good agreements between observed and predicted risks on post-transplant graft survival. All enrolled factors were relatively independent for lower intercorrelation observed in heatmap (all *r* < 0.4, Figure 4).

### Performance of Nomogram Based Alogrithm on Prediction of Prognosis

Efficiency of predictive model was estimated seperately, based on clinical, metabonomic, and combinative factor clusters extracted from nomogram algorithm referred above (Figure 4 and Supplementary Table S7). Meanwhile, performance of these predictive clusters on post-transplant GF was also evaluated in subgroups divided by medical centers. The AUC for prediction of overall graft survival was 0.69 (95%CI: 0.58−0.79), 0.85 (95%CI: 0.75−0.92), and 0.91 (95%CI: 0.83−0.96) for clinical, metabonomic and combinative model (Figure 4 and Supplementary Table S7). Continuous time-dependent AUC of post-transplant GF based on clinical, metabonomic and combinative factors from nomogram algorithm was also evaluated in enrolled patients with extension to 3 years (Figure 4).

Predictive accuracy for clinical cluster (with inclusion of recipient, graft and surgical factors) on post-transplant GF was descended rapidly followed with extended survival time. And the AUC on GF prediction was decreased from 0.81 for 180-day to 0.67 for 3-year graft survival. Compared to clinical clusters, the metabonomic cluster was more stable on GF prediction with lower fluctuation on different time-points (AUC ranged between 0.83 and 0.87). The sensitivity and specificity of clinical-metabonomic model on GF prediction can be reached to 0.93 and 0.81 under the optimal cut-off value (Youden index = 0.74, Supplementary Table S7). By contrast, the Youden index was only 0.32 under the same circumstance for predictive clinical model, with relatively higher sensitivity (0.99) but much lower specificity (0.33). Participation of metabonomic data significantly improved the efficiency of predictive model on post-transplant GF (*P* < 0.01, for AUC comparison between combinative and clinical model, Figure 4 and Supplementary Table S7). And most of the results were also confirmed by internal validation tests conducted in subgroups divided by medical centers (Figure 4 and Supplementary Table S7).

## Discussion

As “last resort” for end-stage liver disease, the quality of LT is affected by multi-factors on donor, recipient, organ, and surgical aspects. More suboptimal organs are put into use to relieve the contradiction between limited organ supply and increasing demands on LT (Tullius and Rabb, 2018). As one of the commonest feature of ECDs, inferior outcomes was observed in patients received severe MaS allografts with more comorbidities, complications and graft failures (Spitzer et al., 2010). As temporary insufficient liver function in shorter PODs, the initial poor function (IPF) was considered to be influenced by donor, recipient, graft and surgical covariates (Hao et al., 2011). Usually, the IPF is reversible by intensive support within 1 month, with similar post-transplant outcomes compared to patients with immediate function (Stockmann et al., 2010). However, the GF risk was amplified in IPF patients by integration with donor MaS (Liu et al., 2020). We speculated that some metabolites as downstream products of biochemical and physiological processes might be responsible for the additional risk of GF caused by MaS donor. Based on metabonomic data from allografts with IPF after LT, we found 1. Metabolites enriched on the pathway of glycerophospholipids metabolism both affected the donor MaS and graft survival; 2. Decrement of molecules including phosphatidylcholine (PC(20:5/16:0), C00157), and phosphatidylethanolamine (PE(20:4/22:6), C00350) were found to be key regulators with responsibility on donor MaS and graft loss; 3. The combinative clinical-metabonomic model (including 10 metabolites and 5 clinical indicators) had improved performance on GF prediction in the following 3 years after LT (AUROC = 0.91). And reliability of this model on prognostic prediction was also confirmed by validation test.

Highly prevalent of donor MaS (>40%) was observed in IPF patients from our study (Table 1). MaS organs suffered more HCV infection and longer time for warm ischemia (*P* < 0.05). Application of MaS allografts was increased over time-period (Supplementary Figure S2). Amount of metabonomic analysis on serum, plasma, urine, liver tissue or even salivar samples from NAFLD/NASH patients in general population were performed to discriminate the suspicious objects, uncover the mechanism and evaluate the efficiency of medical treatment on hepatic steatosis (Gitto et al., 2018; Troisi et al., 2019). However, less metabonomic analysis was performed on grafts discriminated by MaS status before. As we known, the metabonomic change of MaS organs *in vitro* was more complex for higher stress from ischemia-reperfusion injury. Otherwise, most of deceased donors were hospitalized patients with more comorbidities and complications prior to organ donation (Merion et al., 2006). Hence, it is worthy to have metabonomic study to elucidate the metabolic signature for MaS grafts for LT.

In our study, variation on lipid metabolism played a dominant effects role in regulating the hepatic triglyceride content (HTGC) of grafts for LT. PCA analysis revealled patients can be clearly discriminated by donor MaS status (Figure 2). Key molecules was enriched on pathways that related to linoleic acid and glycerophospholipid metabolism. Linoleic acid (LC), as “omega-6 polyunsaturated fatty acid (n-6 PUFA),” is an essential fatty acid only derived from diet, with trade-off relationship to n-3 PUFA *in vivo*. Previous studies found lower n-6:n-3 PUFA ratio might help to ameliorate the ischemia/reperfusion injury via improvement on hepatic microcirculation with potential for clinical implication (Alwayn et al., 2005; Elbadry et al., 2007). Correspondingly, our results confirmed higher n-6 PUFA in MaS grafts on “omics” perspective. Phosphatidylcholine (PC) as antagonist of free cholesterol (FC), was down-regulated, with negative regulation on LC production. Noteworthy, PC was presented as the central hubs to connect the linoleic acid and glycerophospholipid metabolism. Meanwhile, PE was also diminished with more extents (FC = 0.31 and 0.29 vs. 0.45 for PC), with resultant increased PC/PE ratio, indicating relatively mild steatohepatitis in whole grafts (Li et al., 2006;Ling et al., 2012). In addition, increased lysophospholipids (LysoPC) as indication of oxidative stress and proinflammatory status was also involved in pathogenesis of MaS organs. Basically conformed to previous results from NAFLD patients or mice models (Puri et al., 2007; Eisinger et al., 2014). Network analysis revealed the metabonomic changes of tissues from MaS allografts were similar to biopsy tissues from NAFLD/NASH patients.

Six lipid metabolites with hazardous effects on post-transplant graft survival were enriched on the pathway of steroid biosynthesis significantly (*P* < 0.05, Figure 4 and Table 2). This is a novel enriched pathway associated with GF, which was never identified before. As derivatives of cholesterol, steroids were mainly regulated by liver. Steroid derangement might cause NAFLD and inflammation in liver (Charninatan et al., 2019). Concensus on benefits from early withdraw of steroid after LT also implied its potential toxicity for post-transplant prognosis (Lerut et al., 2009; Kimura et al., 2018). Accordingly, our results showed concerns should also be raised on endogenous steroid dysregulation for better post-transplant prognosis.

Lipid played crucial role in determination of complications (EAD, PNF) (Cortes et al., 2014; Faitot et al., 2017) and prognosis (Xu et al., 2015; Tsai et al., 2018) after LT in previous studies. However, less study was focused on MaS related metabolites with simutaneous responsibility for GF occurrence. In our study, intersection was collected between metabolite clusters that related to MaS and GF. The intersected compounds were considered as “bridge” to connect MaS and GF. Finally, the pathway on glycerophospholipid metabolism was significant for MaS related GF (Figure 4).

As major component of cellular membrane, the glycerophospholipid includes collective species of derivative of glycerophosphoric acid (Hermansson et al., 2011). Disturbance on homeostasis of glycerophospholipid might mediate the progression of hepatic Steatosis via enhanced hepatic inflammation (Tanaka et al., 2012; Asimakopoulou et al., 2017). However, glycerophospholipid as connection from donor MaS and post-transplant GF wasn’t reported before. In our study, the PC and PE as key nodes in glycerophospholipid metabolism were only two molecules with negative correlation to inferior prognosis of recipients after LT. And the standardized PC/PE ratio was decreased from 1.5 to 0.76 (*P* < 0.05, Figure 4). Our results indicated the decreased PC/PE ratio and its indicative loss of membrane integrity and severer hepatic inflammation (Li et al., 2006) might be involved in the lethal pathogenesis. The allograft quality might be improved by PC supplement, which was used for NASH patients (Buang et al., 2005). Discrete molecules were mainly belong to lipids and organic acids classes (Table 3) by lower interaction with each other (Figure 4). Organs with extremely higher external substances like Dexamethasone or N-Malonyltryptophan (top 10%) had higher rates of graft failure (62.5%). In addition, all organs with extremely high volume of glucocorticoid residue (top 10%) were grafts from DCD donors. Previous study found inhibitory effects of dexamethasone on initial post-tranplant progression of cell cycle in rats model (Debonera et al., 2003). We speculated that elevated external compounds was indicator for poor graft function on detoxification capability with high probability on GF outcome. Previous studies tried to predict short-term outcomes (EAD, PNF and 3-month mortality) based on molecules obtained from metabonomic (Cortes et al., 2014; Faitot et al., 2017)/lipidomic (Debonera et al., 2003) studies. However, less study was performed to assess the predictive efficiency of metabonomic data on post-transplant outcomes in a dimension with longer follow-up periods. As we known, outcomes varied for profound heterogeneity across individual LT cases. In our study, the integrative model was combination with metabonomic and peri-operative factors related to recipient (height, child-pugh score, EAD), surgery (blood loss) and allograft (steatosis type) based on rigorous alogrithm. Better efficiency on GF prediction was observed for integrative model than clinical model (AUROC = 0.94 vs.0.77, *P* < 0.05). Validation test also confirmed its reliability and availability for outcome prediction (Figure 4). Consistence on time-dependent AUROC (from 0.93 for 180-days to 0.86 for 3-year graft survival) with less attenuation implied its stability on prediction of longterm survival. Metabonomic analysis on allografts plus peri-operative clinical data was effective on prediction of long-term prognosis after LT and worthy for further investigation.

In addition, subjects in our study were selected based on almost 1000 LT cases with IPF occurrence, which guaranteed similar post-transplant liver function for comparability on effects of metabonomic covariates on long-term prognosis. Otherwise, we found the donor MaS exerted its positive effects on GF in maximum by combination with IPF (Liu et al., 2020). And discrimination was also confirmed on metabolome of allografts by MaS status. Selective cases with IPF occurrence might help for better clarification of MaS related mechanism and its connection to GF.

As we known, LT is a systematic engineering with complicated interaction on recipient, donor, graft and surgical factors (Burra et al., 2016). Previous studies tried to build the connection between metabolites and short-term prognosis, but less was referred to long-term outcomes (Cortes et al., 2014; Xu et al., 2015; Faitot et al., 2017; Tsai et al., 2018). We firstly found the alogrithm with integration of metabonomic and peri-operative factors was capable to monitor the long-term prognosis in good accordance. Developed machine perfusion effectively preserved more organs with normal function and expanded the use of sub-optimal organs (Nasralla et al., 2018). Results in our study might provide targets for further MP treatments for improvement of graft quality. The implanted liver (especially for MaS organs) might benefit from perfusates with PC/PE supplement.

Limitation of our study should also be addressed. Potential bias on comparison was inevitable for heterogeneities on length of WIT, HCV prevalence and follow-up duration between MaS and non-MaS groups. Otherwise, difference on statistic approaches and measurements from diverse centers might also cause systematic deviation on combined results, although it seemed comparable for patients in these two cohorts (Supplementary Table S2). Recent increased application of MaS allografts (Supplementary Figure S2) and insufficient follow-up might cause underestimation on MaS related mortality. Medication on donor and recipient *per se* might affect the global metabolome as confounder on association between donor MaS and GF. Otherwise, metabolome level was changed to adapt the inner environment after LT. And tissues were obtained before LT, which can’t represent the real metabolic status of implanted allografts *in vivo*. Metabonomic analysis on graft tissues biopsied in fixed post-transplant time might provide in-depth knowledge to build the accurate link between organ metabolites and disease phenotypes. For ethnical reason, an extensive study with pre-designed metabonomic study on post-transplant liver biopsies is in progress based on LT models in rats. Potential candidate metabolites needs to be verified in spite of their stable trend in internal validation. However, this work was limited for less samples collected in clinical LT. In addition, our results also needs further validation in external cohorts. And new-built extended cohort is now in preparation.

## Conclusion

In conclusion, the metabonomic features can be distinguished by allograft MaS status in patients with IPF. Both endogenous steroid biosynthesis or exogenous glucocorticoid residue were responsible for post-transplant GF occurrence. Dysfunction on pathway of glycerophospholipid metabolism was the link to connect donor MaS and final GF. Decreased PC and PE were culprits to exert fatal effects of MaS on organ failure. Integrative prognostic model with combined metabonomic and peri-operative clinical data might help for monitoring the long-term GS after LT. This study uncovered the molecular pathogenic mechanism of MaS on GF based on omics data, provided accurate targets for machine perfusion which might help to improve the graft quality and expand the donor pool.

## Data Availability Statement

All datasets generated for this study are included in the article/Supplementary Material. Original anonymous omics data is available on request from the corresponding author at liuzhengtao@zju.edu.cn.

## Ethics Statement

The studies involving human participants were reviewed and approved by The First Affiliated Hospital of Zhejiang University and Shulan Hospital Affiliated to Zhejiang Shuren University Shulan International Medical College, respectively. The patients/participants provided their written informed consent to participate in this study.

## Author Contributions

ZL and SZ conceived and designed the study. WW, JX, LiZ, and JQ extracted the information. ZL, HZ, SQ, SW, and JY analyzed the data. ZL and LG wrote the manuscript. FZ, SY, HX, LinZ, and SZ reviewed the manuscript. All authors approved the final manuscript for submission.

## Conflict of Interest

The authors declare that the research was conducted in the absence of any commercial or financial relationships that could be construed as a potential conflict of interest.

## Footnotes

1 http://www.hmdb.ca/

2 https://www.metaboanalyst.ca/MetaboAnalyst/home.xhtml

3 https://hmdb.ca

## Supplementary Material

FIGURE S1 Study flow diagram on impact of metabonomic analysis on post-transplant outcomes.

FIGURE S2 Trend on utilization of steatotic allografts followed with operational period and selection procedure in all LT cases. **(A)** Prevalence of MaS and MiS donor utilization in each year. **(B)** Selection procedure in all LT cases.
