# Supplementary material for: Metabonomic Profile of Macrosteatotic Allografts for Orthotopic Liver Transplantation in Patients With Initial Poor Function: Mechanistic Investigation and Prognostic Prediction
Source: Front Cell Dev Biol. 2020 Aug 28;8:826. doi: 10.3389/fcell.2020.00826 (PMC7484052; doi:10.3389/fcell.2020.00826)
Supplement: FIGURE S1 — Study flow diagram on impact of metabonomic analysis on post-transplant outcomes. [file Data_Sheet_1.docx]

**Supplementary material**

**Procedure for sample preparation**

**Chemicals**

All chemicals and solvents were analytical or HPLC grade. Water, methanol, acetonitrile, formic acid were purchased from CNW Technologies GmbH (Düsseldorf, Germany). L-2-chlorophenylalanine was purchased from Shanghai Hengchuang Bio-technology Co., Ltd (Shanghai, China).

**Sample Preparation**

Samples were accurately weighed and transferred to a 1.5-mL Eppendorf tube (19.8±1.9mg for non-MaS, 19.7±1.8mg for MaS samples, all samples ranged between 15 and 25 mg). Two small steel balls were added to the tube. 20 uL of internal standard (composed by 2-chloro-l-phenylalanine in methanol, 0.3 mg/mL) and 80 uL of extraction solvent with methanol /water (4/1, v/v) were added to each sample. Then, samples were stored at -80℃ for 2 min and grinded at 60 HZ for 2 min, ultrasonicated at ambient temperature (25 ℃) for 10 min, and stored at -20 ℃ for 30 min. The extract was centrifuged at 13000 rpm at 4℃ for 15 min. 5 mL of supernatant in a brown and glass vial was dried in a freeze concentration centrifugal dryer. 100 uL of mixture with methanol and water (1/4, vol/vol) were added to each sample, samples were vortexed for 30 s, then placed at 4℃ for 2 min. Samples were centrifuged at 13000 rpm at 4℃ for 5 min. The supernatants from each tube were collected using crystal syringes, filtered through 0.22 um of microfilters and transferred to LC vials. The vials were stored at -80℃ until LC -MS analysis.

**Sample detection**

Dionex Ultimate 3000 RS UHPLC system fitted with Q-Exactive quadrupole-Orbitrap mass spectrometer equipped with heated electrospqray ionization (ESI) source (Thermo Fisher Scientific, Waltham, MA, USA) was used to analyze the metabolic profiling in both ESI

positive and ESI negative ion modes. An ACQUITY UPLC BEH C18 column (1.7 μm, 2.1 × 100 mm) were employed in both positive and negative modes. The binary gradient elution system (A) consisted of water (containing 0.1% of formic acid, v/v) and acetonitrile (containing 0.1% of formic acid, v/v) and separated with the following gradient: 5–20% A over 0–2 min, 20–60% A over 2–4 min, 60–100% A over 4–11 min, the composition was held at 100% A for 2 min, then 13–13.5 min, 100 % to 5 % A, and 13.5–14.5 min holding at 5 % A. The flow rate was 0.4 mL/min and column temperature was 45℃. All samples were kept at 4℃ during the analysis. Injection volume was 5 μL. The mass range was from m/z 66.7 to 1,000.5. The resolution was set at 70,000 for the full MS scans and 35,000 for HCD MS/MS scans. The Collision energy was set at 10, 20 and 40 eV. The parameters for mass spectrometer was set as follows: spray voltage, 3,000 V (+) and 2,500 V (−); sheath

gas flow rate, 45 arbitrary units; auxiliary gas flow rate, 15 arbitrary units; and capillary temperature, 350°C. QCs were injected at regular intervals each 10 samples throughout the analytical run to provide a set of data from which repeatability can be assessed.

Figure S1


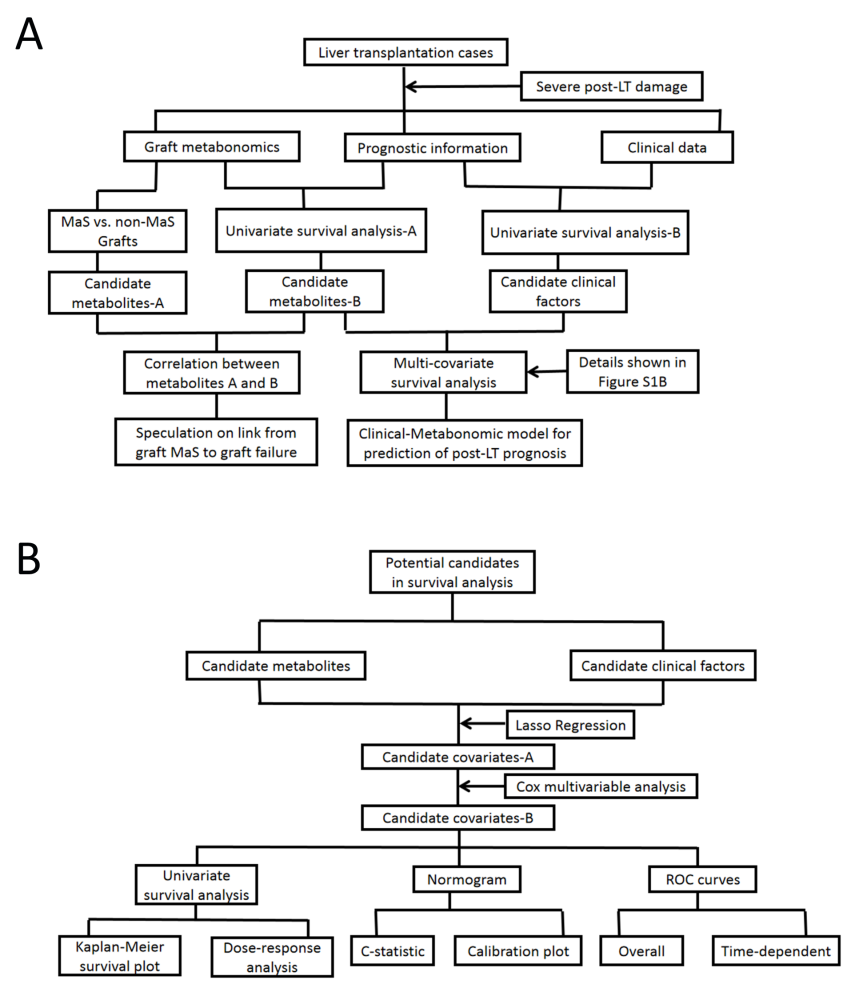


Figure S2


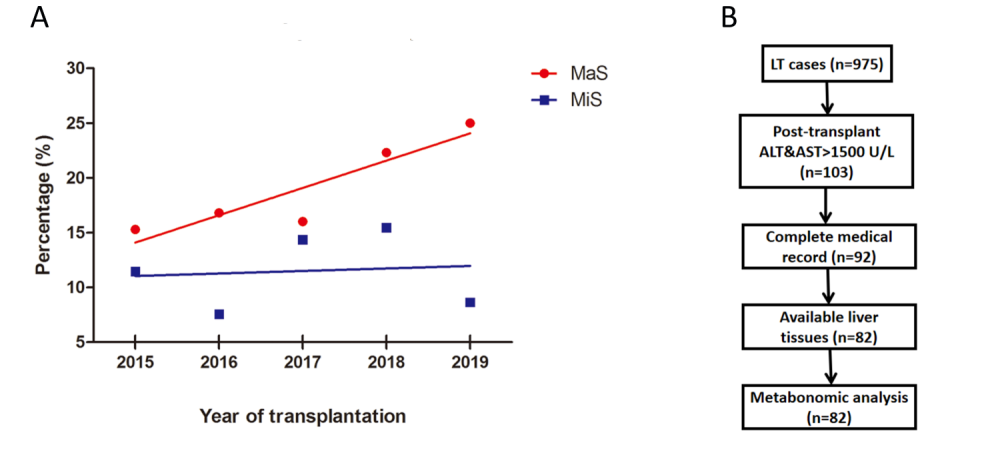


Table S1 Details of software and alogrithm used in statistical analysis

| Statistics | Covariate^a^ & Comparison | Software | Package |
| --- | --- | --- | --- |
| One-way ANOVA test | Clinical univariate | SPSS |  |
| Mann-Whitney U test | Clinical univariate | SPSS |  |
| Dose-response analysis | Clinical/metabonomic | Stata | glst |
| Chi-square test | Clinical univariate | SPSS |  |
| One-way ANOVA test | Metabnomic univariate | R | stats basic |
| Mann-Whitney U test | Metabnomic univariate | R | stats basic |
| Cox proportional-hazards regression test | Clinical/metabonomic univariate | R | survival |
| Multi-covariate cox proportional-hazards regression test | Clinical+metabonomic | R | rms |
| Lasso regression test | Clinical/metabonomic | R | glmnet |
| AUROC comparison | Clinical vs. metabonomic vs. combinative clusters | Medcalc |  |
| ROC curves | Clinical,metabonomic and combinative clusters | Medcalc |  |
| Time-dependent ROC-curves | Clinical,metabonomic and combinative clusters | R | timeROC |
| Correlation heatmaps | Clinical+metabonomic | R | ggcorrplot |
| P-value compared between time-dependent ROC-curves | Clinical,metabonomic and combinative clusters | R | timeROC |
|  |  |  |  |

^a^ Covariates used in assessment can be divided into clinical and metabonomic related covariates.

Table S2 Summary of Clinical Information for Transplant Cases Categorized by Medical Centers

| Covariates | Cohort A | Cohort B | P-value^a^ |
| --- | --- | --- | --- |
| Number(%) | 33(40.2) | 49(59.8) | NA |
| Recipient factor(R) |  |  |  |
| Age(R, years) | 52.0(41.0-56.5) | 47.3(40.8-54.1) | 0.15 |
| Gender(R, M%) | 29(87.9) | 41(83.7) | 0.60 |
| BMI(R, kg/m^2^) | 23.1±3.5 | 23.7±2.7 | 0.40 |
| Blood Type(R) |  |  | 0.42 |
| A-type n(%) | 12(36.4) | 21(42.9) |  |
| B-type n(%) | 3(9.1) | 7(14.3) |  |
| O-type n(%) | 17(51.5) | 17(34.7) |  |
| AB-type n(%) | 1(3.0) | 4(8.2) |  |
| Diabetes(R, N, %) | 5(15.2) | 5(10.2) | 0.50 |
| Pre-operative AFP(R, ng/ml) | 67.6(6.4-1193.5) | 11.4(4.7-80.9) | 0.02 |
| HBV infectors(R, N, %) | 26(78.8) | 37(75.6) | 0.73 |
| MELD score(R) | 34.0(29.5-40.0) | 31.0(23.5-40.0) | 0.10 |
| Child–Pugh score(R) | 10.0(9.0-11.0) | 11.0(9.5-12.0) | 0.19 |
| Donor factor(D) |  |  |  |
| Age(D, years) | 47.0(36.0-52.0) | 44.0(31.5-51.5) | 0.24 |
| Gender (D, M, %) | 28 (84.8) | 41 (83.7) | 0.89 |
| BMI (D, kg/m^2^) | 23.2±2.3 | 23.3±2.9 | 0.96 |
| Blood type (D) |  |  | 0.17 |
| A-type n (%) | 12 (36.4) | 15 (30.6) |  |
| B-type n (%) | 2 (6.1) | 10 (20.4) |  |
| O-type n (%) | 16 (48.5) | 16 (32.7) |  |
| AB-type n (%) | 3 (9.1) | 8 (16.3) |  |
| HBV infectors (D,N,%) | 7 (21.2) | 4 (8.2) | 0.09 |
| HCV infectors (D,N,%) | 4 (12.1) | 2 (4.1) | 0.17 |
| Pre-donation blood test (D) |  |  |  |
| D-Potasium (mmol/L) | 3.6 (3.2-3.8) | 4.1(3.7-4.6) | <0.01 |
| D-Sodium (mmol/L) | 145.9 (138.5-151.5) | 146.0 (139.0-154.0) | 0.52 |
| D-ALT (U/L) | 42.0(24.8-67.4) | 39.4 (25.5-66.5) | 0.90 |
| D-TB (umol/L) | 16.0(9.8-22.0) | 17.9(11.1-28.8) | 0.18 |
| D-CR (μmol/L) | 80.0(54.0-103.5) | 95.0(63.1-192.0) | 0.05 |
| D-BUN (mmol/L) | 7.4(5.4-9.5) | 8.9(5.1-12.4) | 0.32 |
| Donation type (DBD/DCD/DBCD) |  |  | 0.29 |
| DBD (N,%) | 10 (30.3) | 12 (24.5) |  |
| DCD (N,%) | 20 (60.6) | 26 (53.1) |  |
| DBCD (N,%) | 3 (9.1) | 11 (22.4) |  |
| Cause of Death (TBI/Stroke/Others) | 20/10/3 | 21/26/2 | 0.11 |
| Graft factor(G) |  |  |  |
| CIT(min) | 652.0(586.5-694.0) | 651.0(498.5-775.5) | 0.94 |
| WIT(min) | 5.0(2.0-10.0) | 9.0(4.0-12.0) | 0.08 |
| Surgery(S) |  |  |  |
| Indication for LT |  |  | 0.08 |
| Liver Cirrhosis n (%) | 14(42.4) | 21(42.9) |  |
| Liver Failure n (%) | 11 (33.3) | 6(12.2) |  |
| PBC/PSC n (%) | 1(3.0) | 3(6.1) |  |
| Liver Cancer n (%) | 23(69.7) | 13(26.5) |  |
| Others n (%) | 1(3.0) | 2(4.1) |  |
| Post-LT Peak TB level (mg/dL) | 179.0(81.5-349.0) | 228.0(154.2-408.5) | 0.06 |
| Post-LT Peak ALT Level (IU/L) | 2977.0(2515.0-3655.0) | 2235.0(1834.5-3019.5) | <0.01 |
| Post-LT Peak AST level (IU/L) | 7649.0(5568.0-9826.5) | 5514.0(4018.5-8812.5) | 0.11 |
| Blood Loss (ml) | 2000 (800-2500) | 1500 (900-2000) | 0.31 |
| Surgical Duration (mins) | 294.0(265.0-329.5) | 320.0 (285.5-378.5) | 0.09 |
|  |  |  |  |
| * represented significant difference across different groups; a represented  ^a^ comparison was performed by one-way ANOVA for quantitative data in symmetrical distribution; by Mann-Whitney U test for quantitative data in asymmetrical distribution; by Chi-square test for count data.  Abbreviations:D, donor; DBCD, donation after brain and cardiac death; DBD, donation after brain death; DCD, donation after cardiac death; G, graft; HBV, hepatitis B virus; HCV, hepatitis C virus; LT, liver transplantation; M, male; MELD, model for end-stage liver disease; PBC, primary biliary cholangitis; primary sclerosing cholangitis; R, recipient; RBC, red blood cell; TB, total bilirubin; TBI, traumatic brain injuries. | | | |

Table S3 Pathway analysis based on enriched differential metabolites classified by MaS status

|  | Total | Expected | Hits | Raw p | Impact |
| --- | --- | --- | --- | --- | --- |
| Linoleic acid metabolism | 5 | 0.27097 | 2 | 0.026081 | 1 |
| Glycerophospholipid metabolism | 36 | 1.951 | 5 | 0.041475 | 0.24464 |
| alpha-Linolenic acid metabolism | 13 | 0.70452 | 2 | 0.15382 | 0.33333 |
| Glycosylphosphatidylinositol (GPI)-anchor biosynthesis | 14 | 0.75871 | 2 | 0.17342 | 0.00399 |
| Biosynthesis of unsaturated fatty acids | 36 | 1.951 | 3 | 0.30926 | 0 |
| Taurine and hypotaurine metabolism | 8 | 0.43355 | 1 | 0.36031 | 0 |
| Phenylalanine metabolism | 10 | 0.54194 | 1 | 0.42813 | 0 |
| Glutathione metabolism | 28 | 1.5174 | 2 | 0.45423 | 0.05158 |
| Porphyrin and chlorophyll metabolism | 30 | 1.6258 | 2 | 0.49078 | 0.11353 |
| Arginine biosynthesis | 14 | 0.75871 | 1 | 0.54316 | 0 |
| Arachidonic acid metabolism | 36 | 1.951 | 2 | 0.59091 | 0 |
| Pentose and glucuronate interconversions | 18 | 0.97548 | 1 | 0.63528 | 0.14062 |
| Tryptophan metabolism | 41 | 2.2219 | 2 | 0.66279 | 0 |
| Sphingolipid metabolism | 21 | 1.1381 | 1 | 0.69207 | 0.26978 |
| Lysine degradation | 25 | 1.3548 | 1 | 0.75442 | 0.04695 |
| Galactose metabolism | 27 | 1.4632 | 1 | 0.78074 | 0.06143 |
| Alanine, aspartate and glutamate metabolism | 28 | 1.5174 | 1 | 0.79283 | 0.02163 |
| Phosphatidylinositol signaling system | 28 | 1.5174 | 1 | 0.79283 | 0.09747 |
| Inositol phosphate metabolism | 30 | 1.6258 | 1 | 0.81508 | 0.0777 |
| Steroid biosynthesis | 42 | 2.2761 | 1 | 0.90676 | 0 |
| Tyrosine metabolism | 42 | 2.2761 | 1 | 0.90676 | 0.12972 |
| Primary bile acid biosynthesis | 46 | 2.4929 | 1 | 0.92588 | 0 |
| Steroid hormone biosynthesis | 85 | 4.6065 | 2 | 0.95283 | 0.14466 |
| Purine metabolism | 65 | 3.5226 | 1 | 0.9753 | 0 |
|  |  |  |  |  |  |

Table S4 Pathway analysis based on enriched differential metabolites classified by occurrence of graft failure

|  | Total | Expected | Hits | Raw p | Impact |
| --- | --- | --- | --- | --- | --- |
| Steroid biosynthesis | 42 | 2.249 | 6 | 0.021836 | 0.09078 |
| Pentose phosphate pathway | 22 | 1.1781 | 3 | 0.10949 | 0.31484 |
| Glycerophospholipid metabolism | 36 | 1.9277 | 4 | 0.12254 | 0.23527 |
| alpha-Linolenic acid metabolism | 13 | 0.69613 | 2 | 0.15086 | 0 |
| Linoleic acid metabolism | 5 | 0.26774 | 1 | 0.24084 | 0 |
| Arachidonic acid metabolism | 36 | 1.9277 | 3 | 0.30273 | 0.1166 |
| Pyrimidine metabolism | 39 | 2.0884 | 3 | 0.34794 | 0.14269 |
| Vitamin B6 metabolism | 9 | 0.48194 | 1 | 0.39143 | 0.07843 |
| Caffeine metabolism | 10 | 0.53548 | 1 | 0.42421 | 0 |
| Biotin metabolism | 10 | 0.53548 | 1 | 0.42421 | 0.2 |
| Glycosylphosphatidylinositol (GPI)-anchor biosynthesis | 14 | 0.74968 | 1 | 0.53876 | 0.00399 |
| Arginine biosynthesis | 14 | 0.74968 | 1 | 0.53876 | 0.06091 |
| Nicotinate and nicotinamide metabolism | 15 | 0.80323 | 1 | 0.56368 | 0 |
| Glycerolipid metabolism | 16 | 0.85677 | 1 | 0.58728 | 0 |
| Arginine and proline metabolism | 38 | 2.0348 | 2 | 0.61424 | 0.11063 |
| Terpenoid backbone biosynthesis | 18 | 0.96387 | 1 | 0.63074 | 0.11429 |
| Pentose and glucuronate interconversions | 18 | 0.96387 | 1 | 0.63074 | 0.14062 |
| Pantothenate and CoA biosynthesis | 19 | 1.0174 | 1 | 0.65075 | 0.02857 |
| Tryptophan metabolism | 41 | 2.1955 | 2 | 0.65619 | 0.00202 |
| Fructose and mannose metabolism | 20 | 1.071 | 1 | 0.66968 | 0.00311 |
| Citrate cycle (TCA cycle) | 20 | 1.071 | 1 | 0.66968 | 0.04412 |
| Sphingolipid metabolism | 21 | 1.1245 | 1 | 0.6876 | 0.02434 |
| beta-Alanine metabolism | 21 | 1.1245 | 1 | 0.6876 | 0.10448 |
| Pyruvate metabolism | 22 | 1.1781 | 1 | 0.70456 | 0.0311 |
| Lysine degradation | 25 | 1.3387 | 1 | 0.75017 | 0.04695 |
| Glycolysis / Gluconeogenesis | 26 | 1.3923 | 1 | 0.76376 | 0 |
| Folate biosynthesis | 27 | 1.4458 | 1 | 0.77663 | 0.09924 |
| Glutathione metabolism | 28 | 1.4994 | 1 | 0.7888 | 0 |
| Inositol phosphate metabolism | 30 | 1.6065 | 1 | 0.81122 | 0 |
| Glyoxylate and dicarboxylate metabolism | 32 | 1.7135 | 1 | 0.83128 | 0 |
| Biosynthesis of unsaturated fatty acids | 36 | 1.9277 | 1 | 0.86529 | 0 |
| Tyrosine metabolism | 42 | 2.249 | 1 | 0.90401 | 0 |
| Purine metabolism | 65 | 3.4806 | 1 | 0.97415 | 0.0145 |
| Steroid hormone biosynthesis | 85 | 4.5516 | 1 | 0.99188 | 0.00522 |
|  |  |  |  |  |  |

Table S5 Pathway analysis based on enriched differential metabolites classified by occurrence of MaS related graft failure

|  | Total | Expected | Hits | Raw p | Impact |
| --- | --- | --- | --- | --- | --- |
| Glycerophospholipid metabolism | 36 | 0.092903 | 2 | 0.0030573 | 0.19895 |
| Linoleic acid metabolism | 5 | 0.012903 | 1 | 0.012853 | 0 |
| alpha-Linolenic acid metabolism | 13 | 0.033548 | 1 | 0.03316 | 0 |
| Glycosylphosphatidylinositol (GPI)-anchor biosynthesis | 14 | 0.036129 | 1 | 0.035677 | 0.00399 |
| Pentose and glucuronate interconversions | 18 | 0.046452 | 1 | 0.045692 | 0.14062 |
| Lysine degradation | 25 | 0.064516 | 1 | 0.063032 | 0.04695 |
| Arachidonic acid metabolism | 36 | 0.092903 | 1 | 0.0898 | 0 |
|  |  |  |  |  |  |

Table S6 Dose-response effects of continuous clinical-metabonomic factors on post-transplant graft failure

| Item | Regression Model/  HR(95%CI)^a^ | Graft failure (%) | P1b for non-linearity  /P2c for significance |
| --- | --- | --- | --- |
| HMDB0000052 | GLS |  | 0.090/0.009 |
| Q1 | Ref | 6.3 |  |
| Q2 | 1.09(1.02-1.16) | 41.3 |  |
| Q3 | 1.16(1.04-1.29) | 25.0 |  |
| Q4 | 1.37(1.08-1.73) | 52.9 |  |
| Q5 | 1.09(1.02-1.16) | 56.3 |  |
| HMDB0008232 | GLS |  | 0.355/0.004 |
| Q1 | Ref | 52.9 |  |
| Q2 | 0.94(0.90-0.98) | 31.3 |  |
| Q3 | 0.85(0.77-0.95) | 31.3 |  |
| Q4 | 0.79(0.67-0.93) | 52.9 |  |
| Q5 | 0.69(0.54-0.89) | 12.5 |  |
| HMDB0013288 | GLS |  | 0.242/0.285 |
| Q1 | Ref | 43.8 |  |
| Q2 | 0.99(0.98-1.01) | 29.4 |  |
| Q3 | 0.99(0.97-1.01) | 56.3 |  |
| Q4 | 0.98(0.94-1.02) | 23.5 |  |
| Q5 | 0.92(0.80-1.07) | 31.3 |  |
| HMDB0015364 | GLS |  | 0.385/0.040 |
| <0.01 | Ref | 31.7 |  |
| 0.01-1 | 1.01(0.99-1.02) | 37.5 |  |
| 1.01-10 | 1.01(1.00-1.02) | 33.3 |  |
| >10 | 1.21(1.06-1.39) | 55.6 |  |
| HMDB0029069 | RCS |  | 0.019/NA |
| Q1 | Ref | 25.0 |  |
| Q2 | 1.22(1.07-1.40) | 17.6 |  |
| Q3 | 1.49(1.14-1.95) | 37.5 |  |
| Q4 | 1.52(1.18-1.95) | 52.9 |  |
| Q5 | 1.42(1.19-1.69) | 50.0 |  |
| HMDB0038029 | RCS |  | 0.001/NA |
| Q1 | Ref | 31.3 |  |
| Q2 | 1.07(1.02-1.12) | 35.3 |  |
| Q3 | 1.29(1.07-1.55) | 37.5 |  |
| Q4 | 1.16(0.97-1.39) | 58.8 |  |
| Q5 | 0.82(0.69-0.97) | 18.8 |  |
| HMDB0038943 | GLS |  | 0.134/<0.001 |
| Q1 | Ref | 56.3 |  |
| Q2 | 0.97（0.96-0.99） | 35.3 |  |
| Q3 | 0.93（0.90-0.97） | 50.0 |  |
| Q4 | 0.87（0.80-0.94） | 35.3 |  |
| Q5 | 0.63（0.49-0.82） | 6.3 |  |
| HMDB0039500 | GLS |  | 0.176/<0.001 |
| <0.009 | Ref | 32.3 |  |
| 0.010-0.099 | 1.01(1.00-1.02) | 36.8 |  |
| 0.100-0.499 | 1.11(1.05-1.17) | 33.3 |  |
| >0.500 | 1.48(1.21-1.81) | 62.5 |  |
| HMDB0060282 | GLS |  | 0.218/0.002 |
| Q1 | Ref | 43.8 |  |
| Q2 | 0.74(0.61-0.90) | 52.9 |  |
| Q3 | 0.71(0.56-0.88) | 43.8 |  |
| Q4 | 0.67(0.52-0.86) | 17.6 |  |
| Q5 | 0.62(0.45-0.84) | 25.0 |  |
| HMDB0114818 | RCS |  | 0.040/NA |
| Q1+Q2 | Ref | 33.3 |  |
| Q3 | 1.15(1.00-1.32) | 18.8 |  |
| Q4 | 1.31(1.01-1.69) | 47.1 |  |
| Q5 | 1.19(1.00-1.42) | 43.8 |  |
| Blood loss/1000 (ml) | GLS |  | 0.102/0.001 |
| 0-0.80 | Ref | 19.0 |  |
| 0.81-1.00 | 1.09(1.03-1.14) | 33.3 |  |
| 1.50-2.00 | 1.13(1.05-1.22) | 43.8 |  |
| 2.00-2.50 | 1.18(1.07-1.30) | 40.0 |  |
| 3.00-8.10 | 1.38(1.14-1.69) | 53.8 |  |
| R-Child-pugh-score | GLS |  | 0.671/<0.001 |
| 5-9 | Ref | 21.1 |  |
| 10 | 1.29(1.13-1.46) | 42.1 |  |
| 11 | 1.35(1.16-1.58) | 40.0 |  |
| 12 | 1.42(1.19-1.71) | 21.4 |  |
| 13-14 | 1.52(1.22-1.88) | 70.0 |  |
| R-height (cm) | GLS |  | 0.116/0.427 |
| <165.0 | Ref | 36.0 |  |
| 165.1-168.0 | 0.97(0.90-1.05) | 60.0 |  |
| 168.1-171.0 | 0.96(0.88-1.06) | 25.0 |  |
| 171.1-175.0 | 0.95(0.84-1.07) | 44.4 |  |
| >175.0 | 0.93(0.79-1.11) | 23.1 |  |
|  |  |  |  |

^a^ HR represented the risk on GF for compariation between corresponded and baseline part;

^b^ P1 represented the significance of linearity by GLS model;

^C^ P2 represented the significance linear association between candidate covariates and GF by GLS model.

Abbreviations: GF, graft ratio; GLS,generalized least squares; HR,hazard ratio; Q, quintile; R, recipient; RCS,restricted cubic splines.

Table S7 Peformance of different algorithmic models on prediction of graft failure

| Model | AUC (95%CI) | P-value | | | Sensitivity | Specificity | Youden index |
| --- | --- | --- | --- | --- | --- | --- | --- |
|  |  | P1^a^ | P2^b^ | P3^c^ |  |  |  |
| **All patients** |  |  |  |  |  |  |  |
| Overall GS |  |  |  |  |  |  |  |
| Combinative | 0.91(0.83-0.96) |  | 0.04 | <0.01 | 0.93 | 0.81 | 0.74 |
| Metabonomic | 0.85(0.75-0.92) | 0.04 |  | 0.03 | 0.87 | 0.79 | 0.66 |
| Clinical | 0.69(0.58-0.79) | <0.01 | 0.03 |  | 0.99 | 0.33 | 0.32 |
| 180d-GS |  |  |  |  |  |  |  |
| Combinative | 0.93(0.86-0.98) |  | 0.02 | 0.02 | 0.93 | 0.87 | 0.80 |
| Metabonomic | 0.84(0.74-0.93) | 0.02 |  | 0.69 | 0.86 | 0.84 | 0.70 |
| Clinical | 0.81(0.71-0.90) | 0.02 | 0.69 |  | 0.78 | 0.65 | 0.43 |
| 1 year-GS |  |  |  |  |  |  |  |
| Combinative | 0.93(0.86-0.99) |  | 0.12 | 0.01 | 0.90 | 0.89 | 0.79 |
| Metabonomic | 0.87(0.79-0.96) | 0.12 |  | 0.26 | 0.79 | 0.91 | 0.70 |
| Clinical | 0.79(0.68-0.90) | 0.01 | 0.26 |  | 0.72 | 0.64 | 0.36 |
| 2 year-GS |  |  |  |  |  |  |  |
| Combinative | 0.87(0.75-0.98) |  | 0.50 | 0.04 | 0.93 | 0.87 | 0.80 |
| Metabonomic | 0.83(0.70-0.96) | 0.50 |  | 0.25 | 0.77 | 0.88 | 0.65 |
| Clinical | 0.69(0.65-0.90) | 0.04 | 0.25 |  | 0.90 | 0.54 | 0.44 |
| 3 year-GS |  |  |  |  |  |  |  |
| Combinative | 0.86(0.74-0.98) |  | 0.68 | 0.04 | 0.87 | 0.85 | 0.72 |
| Metabonomic | 0.84(0.70-0.97) | 0.68 |  | 0.19 | 0.81 | 0.84 | 0.65 |
| Clinical | 0.67(0.49-0.86) | 0.04 | 0.19 |  | 0.94 | 0.45 | 0.39 |
| **Cohort A** |  |  |  |  |  |  |  |
| Overall GS |  |  |  |  |  |  |  |
| Combinative | 0.92(0.82-0.98) |  | 0.03 | <0.01 | 0.96 | 0.81 | 0.77 |
| Metabonomic | 0.84(0.72-0.92) | 0.03 |  | 0.07 | 0.80 | 0.78 | 0.58 |
| Clinical | 0.67(0.54-0.79) | <0.01 | 0.07 |  | 1.00 | 0.30 | 0.30 |
| 180d-GS |  |  |  |  |  |  |  |
| Combinative | 0.93(0.86-0.98) |  | 0.02 | 0.02 | 0.91 | 0.86 | 0.77 |
| Metabonomic | 0.84(0.74-0.94) | 0.02 |  | 0.51 | 0.82 | 0.84 | 0.66 |
| Clinical | 0.78(0.67-0.90) | 0.02 | 0.51 |  | 1.00 | 0.40 | 0.40 |
| 1 year-GS |  |  |  |  |  |  |  |
| Combinative | 0.94(0.88-0.99) |  | 0.09 | 0.01 | 0.92 | 0.88 | 0.80 |
| Metabonomic | 0.88(0.79-0.98) | 0.09 |  | 0.19 | 0.92 | 0.76 | 0.68 |
| Clinical | 0.77(0.63-0.90) | 0.01 | 0.19 |  | 1.00 | 0.34 | 0.34 |
| 2 year-GS |  |  |  |  |  |  |  |
| Combinative | 0.88(0.77-0.99) |  | 0.55 | 0.03 | 0.88 | 0.88 | 0.76 |
| Metabonomic | 0.85(0.69-0.99) | 0.55 |  | 0.20 | 0.88 | 0.75 | 0.63 |
| Clinical | 0.65(0.45-0.85) | 0.03 | 0.2 |  | 1.00 | 0.35 | 0.35 |
| 3 year-GS |  |  |  |  |  |  |  |
| Combinative | 0.88(0.77-0.99) |  | 0.55 | 0.03 | 0.88 | 0.88 | 0.76 |
| Metabonomic | 0.85(0.69-0.99) | 0.55 |  | 0.20 | 0.85 | 0.74 | 0.59 |
| Clinical | 0.65(0.45-0.85) | 0.03 | 0.20 |  | 0.96 | 0.33 | 0.29 |
| **Cohort B** |  |  |  |  |  |  |  |
| Overall GS |  |  |  |  |  |  |  |
| Combinative | 0.90(0.80-0.96) |  | 0.64 | <0.01 | 0.89 | 0.83 | 0.72 |
| Metabonomic | 0.89(0.78-0.95) | 0.64 |  | <0.01 | 0.86 | 0.84 | 0.70 |
| Clinical | 0.61(0.48-0.73) | <0.01 | <0.01 |  | 0.61 | 0.62 | 0.23 |
| 180d-GS |  |  |  |  |  |  |  |
| Combinative | 0.91(0.84-0.98) |  | 0.08 | 0.02 | 0.80 | 0.89 | 0.69 |
| Metabonomic | 0.85(0.76-0.95) | 0.08 |  | 0.26 | 0.84 | 0.83 | 0.67 |
| Clinical | 0.76(0.64-0.88) | 0.02 | 0.26 |  | 0.72 | 0.65 | 0.37 |
| 1 year-GS |  |  |  |  |  |  |  |
| Combinative | 0.93(0.86-0.99) |  | 0.21 | 0.01 | 0.78 | 0.94 | 0.72 |
| Metabonomic | 0.89(0.81-0.97) | 0.21 |  | 0.11 | 0.78 | 0.89 | 0.67 |
| Clinical | 0.76(0.63-0.89) | 0.01 | 0.11 |  | 0.70 | 0.61 | 0.31 |
| 2 year-GS |  |  |  |  |  |  |  |
| Combinative | 0.89(0.79-0.99) |  | 0.91 | 0.04 | 0.75 | 0.94 | 0.69 |
| Metabonomic | 0.89(0.79-0.99) | 0.91 |  | 0.07 | 0.79 | 0.89 | 0.68 |
| Clinical | 0.70(0.53-0.87) | 0.04 | 0.07 |  | 0.86 | 0.43 | 0.29 |
| 3 year-GS |  |  |  |  |  |  |  |
| Combinative | 0.88(0.77-0.98) |  | 0.71 | 0.04 | 0.76 | 0.92 | 0.68 |
| Metabonomic | 0.89(0.78-0.99) | 0.71 |  | 0.06 | 0.76 | 0.89 | 0.65 |
| Clinical | 0.68(0.50-0.86) | 0.04 | 0.06 |  | 0.80 | 0.89 | 0.69 |
|  |  |  |  |  |  |  |  |

Combinative model included both clinical and metabonomic covariates from nomogram alogrithm;

Metabonomic model included only metabonomic covariates from nomogram alogrithm;

Clinical model included only clinical covariates from nomogram alogrithm.

^a^P1 represented the significance compared between combinative and metabonomic models;

^b^P2 represented the significance compared between combinative and clinical models;

^c^P3 represented the significance compared between metabonomic and clinical models.

Abbreviations: AUC, area under the curve; GS, graft survival.
